# Fisetin attenuates oxidative and haemolytic damage induced by quinuclidine derivatives in human erythrocytes

**DOI:** 10.2478/aiht-2026-77-4134

**Published:** 2026-09-25

**Authors:** Lucija Marcelić, Lea Malezan, Katja Vuković, Maja Katalinić, Antonio Zandona

**Affiliations:** Institute for Medical Research and Occupational Health, Division of Toxicology, Zagreb, Croatia

**Keywords:** antioxidative action, cholinesterases, flavonoids, red blood cells, antioksidacijska aktivnost, crvene krvne stanice, flavonoidi, kolinesteraza, zaštita

## Abstract

Quinuclidine derivatives are potent bioactive compounds investigated as cholinesterase inhibitors with potential intravenous therapeutic application. However, the cytotoxic properties of some, particularly those linked to oxidative stress, pose significant safety concerns. This study aimed to evaluate if flavonoid fisetin could protect cells from such toxic effects. Erythrocytes were chosen due to high susceptibility to reactive oxygen species (ROS) and well-characterised endogenous antioxidant system, making them a sensitive and physiologically relevant model. Over 24 h, human erythrocytes were exposed to QOH-C12–16 or QNOH-C12–16 derivatives to establish their 25, 50, and 75 % inhibitory concentrations (IC_25_, IC_50_, and IC_75_, respectively). As the compounds with the C12 side alkyl chain showed no significant haemolytic effects, they were excluded from further experimentation. In the next steps, we assessed the protective effects of fisetin (1, 10, 100 μmol/L) against quinuclidine-induced haemolysis over 24 h and against reactive oxygen species (ROS) generation and potential glutathione (GSH) and superoxide dismutase (SOD) depletion over 4 h. Fisetin at 10 and 100 μmol/L significantly reduced ROS to near-control levels, restored SOD activity, and decreased haemolysis by 10–40 %, most effectively in cells exposed to quinuclidines at IC_50_. However, GSH measurements in combined treatment were not reliable, as fisetin exhibited autofluorescence, but single quinuclidine exposure showed no significant effects on GSH levels. In summary, fisetin showed a promising potential to make quinuclidine intravenous therapies safer.

## Abstract (hr)

Derivati kinuklidina potentni su bioaktivni spojevi koji se istražuju kao inhibitori kolinesteraza s mogućom intravenskom terapijskom primjenom. Međutim, uočena citotoksičnost pojedinih derivata, osobito ona povezana s oksidacijskim stresom, značajan je sigurnosni problem. Cilj ovoga istraživanja bio je procijeniti može li flavonoid fisetin zaštititi stanice od takvih toksičnih učinaka. Eritrociti su odabrani zbog svoje visoke osjetljivosti na reaktivne kisikove vrste (ROS) i dobro okarakteriziranoga endogenog antioksidacijskog sustava, što ih čini osjetljivim i fiziološki relevantnim modelom. Tijekom 24 sata ljudski eritrociti bili su izloženi derivatima QOH-C12–16 ili QNOH-C12–16 kako bi se odredile njihove 25, 50 i 75 %-tne inhibitorne koncentracije (IC_25_, IC_50_ i IC_75_). Budući da spojevi s C12 bočnim alkilnim lancem nisu pokazali značajne hemolitičke učinke, isključeni su iz daljnjih pokusa. U sljedećim smo koracima procijenili zaštitne učinke fisetina (1, 10 i 100 μmol/L) protiv hemolize izazvane kinuklidinima tijekom 24 sata te protiv stvaranja reaktivnih kisikovih vrsta (ROS) i mogućeg smanjenja razine glutationa (GSH) i aktivnosti superoksid-dismutaze (SOD) tijekom 4 sata. Fisetin je pri koncentracijama od 10 i 100 μmol/L značajno smanjio razinu ROS-a na vrijednosti bliske kontrolnima, obnovio aktivnost SOD-a i smanjio hemolizu 10 – 40 %, i to najučinkovitije u stanicama izloženima kinuklidinima pri IC_50_. Međutim, mjerenja GSH-a u kombiniranom tretmanu nisu bila pouzdana jer je fisetin autofluorescirao, dok pojedinačno izlaganje kinuklidinima nije imalo značajan učinak na razinu GSH-a. Zaključno, fisetin je pokazao obećavajući potencijal za povećanje sigurnosti intravenskih terapija temeljenih na kinuklidinima.

Quinuclidine-based compounds have been investigated as potent acetylcholinesterase (AChE) inhibitors (1,2,3,4), particularly in the context of treating neurodegenerative disorders such as Alzheimer's disease thanks to their selectivity for binding to the AChE active site gorge (5) and capacity to reduce cholinergic deficit central to the Alzheimer's pathology (4). These include derivatives designed and synthetised to improve pharmacokinetics and delivery to the central nervous system (2) like *N*-alkyl quaternary quinuclidine derivatives (Q(N)OH), which have shown promising enzyme inhibition, especially when functionalised with alcohol or oxime groups (1, 6). Structural modifications, including alkylation and oxime derivatisation, employed to optimise binding to both catalytic and peripheral anionic sites of AChE, have been reported to improve their potency and duration of action (1, 2). Such structural features are also investigated for dual-target activity in modulating amyloid aggregation in Alzheimer's disease (3). However, the therapeutic application of these compounds, most notably of derivatives with long alkyl chains (C12–16), could be compromised by their cytotoxicity, leading to oxidative damage in hepatic and neuronal cells in the micromolar concentration range (1, 6, 7).

Furthermore, if given intravenously, blood components are directly exposed to the drug, which may lead to haemolysis (8,9,10,11,12,13,14,15). Due to the absence of mitochondria and the nucleus, erythrocytes cannot upregulate antioxidant defences through transcriptional responses. Instead, they rely on a finite supply of antioxidant enzymes and glutathione to neutralise reactive oxygen species (ROS) (16), and when ROS production overwhelms these defences, irreversible damage occurs (17).

Luckily, this could be minimised by co-administration of antioxidants (18), and polyphenols such as flavonoids, found in various fruits and vegetables have been reported to act as direct radical scavengers, reduce lipid peroxidation, preserve membrane integrity, and protect against oxidative stress (14, 19,20,21). Among them, fisetin (3,3′,4′,7-tetrahydroxyflavone), a naturally occurring flavonol, has shown a great potential in scavenging ROS and protecting cells from oxidative damage implicated in neuroprotection and anti-inflammatory responses *in vitro* and *in vivo* (28,29,30,31). Yet, despite extensive studies of its antioxidant action in various models, its efficacy against membrane-active quinuclidine-based acetylcholinesterase inhibitors has not yet been evaluated. Moreover, the haematological safety of these promising compounds, particularly in the context of intravenous administration, remains poorly understood. Therefore, the aim of this study was to evaluate the cytotoxic effects of Q(N)OH derivatives on erythrocytes and to assess the protective efficacy of fisetin against Q(N)OH-induced oxidative haemolysis. Our ultimate aim was to propose a viable antioxidant strategy to enhance the safety profile of quinuclidine derivatives for potential intravenous therapeutic application.

## MATERIALS AND METHODS

**Figure 1:**
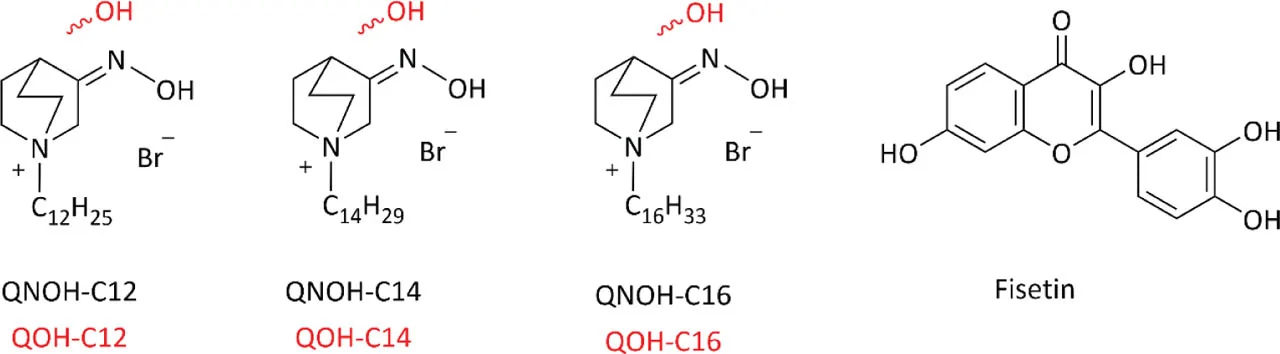
Structure of *N*-alkyl quaternary quinuclidines used in this study

The six investigated Q(N)OHs (QOH-C12–16 and QNOH-C12–16 (Figure 1) were a gift from Professor Ines Primožič (University of Zagreb Faculty of Science, Department of Chemistry, Zagreb, Croatia). They were designed and synthetised as described in previous studies (1, 6). *Tert*-butyl hydrogen peroxide (tBHP), used as oxidative stress inducer, and fisetin, used as antioxidant, were purchased from Sigma-Aldrich (Steinheim, Germany). The Q(N) OHs were prepared as 100 mmol/L stock solutions in dimethyl sulphoxide (DMSO) and fisetin as 10 mmol/L stock solution in methanol and diluted to selected concentrations in phosphate-buffered saline (PBS) prior to experimentation.

### Erythrocyte treatment and haemolysis measurements

Human erythrocytes were isolated from one donor's blood (upon approvals of the Ethics Committee of the Institute for Medical Research and Occupational Health, Zagreb, Croatia Nos. 01-18/23-02-2/1 and 100-21/23-16) following a standard procedure reported elsewhere (32, 33).

**Figure 2:**
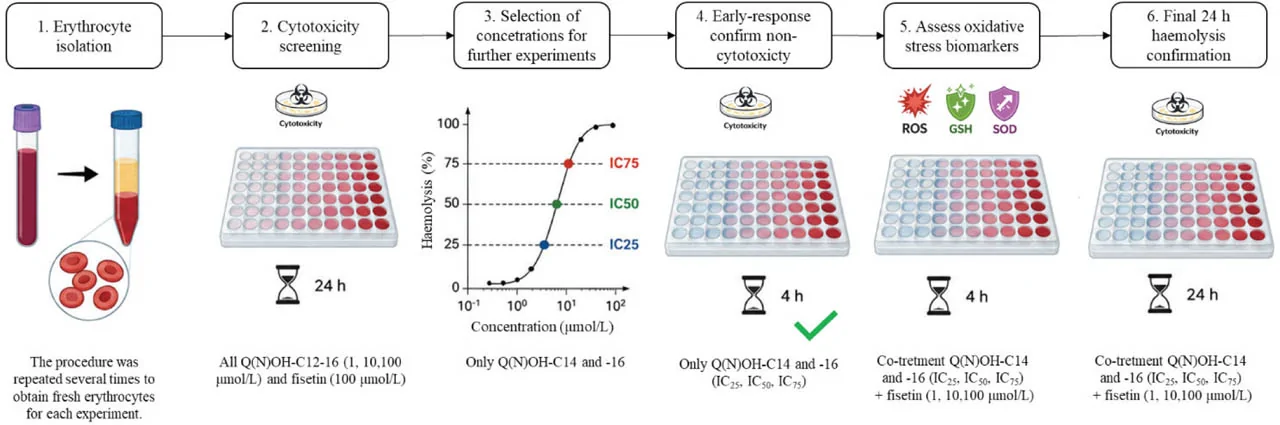
Experimental design for testing quinuclidine derivative and fisetin effects on human erythrocytes

Figure 2 details the experimental design. Briefly, to determine 25 %, 50 %, and 75 % inhibitory concentrations of the Q(N)OHs (IC_25_, IC_50_, and IC_75_, respectively), we diluted the erythrocytes in a PBS to obtain 2 % (*v*/*v*) suspension and exposed them to Q(N)OH concentrations of 0.1–100 μmol/L at 37 °C over for 24 h (gentle stirring applied). Followed centrifugation at 1,000 *g* and 4 °C for 2 min, after which we transferred the supernatants to a clear, flat-bottom 96-well plate to measure released haemoglobin on an Infinite M200PRO plate reader (Tecan Austria GmbH, Salzburg, Austria) at 570 nm. The IC_25_, IC_50_, and IC_75_ for each Q(N)OH were obtained using non-linear regression curves. The determined inhibitory concentrations were then applied to fresh erythrocyte suspensions for 4 h to check if these concentrations would induce extensive haemolysis early. As they did not (see Results), we could proceed to assess early oxidative events, those preceding the final haemolytic outcome.

To see if fisetin would cause haemolysis in erythrocytes, we also exposed the cells to its highest concentration of 100 μmol/L over 24 h and found no adverse effects.

In the final step, we wanted to determine the protective efficiency of fisetin (1, 10, and 100 μmol/L) against the obtained Q(N)OH inhibitory concentrations in a fresh batch of erythrocytes exposed to those compounds for 24 h with or without fisetin. Haemolysis was measured as described above.

Untreated erythrocytes in PBS were used as negative control, and those treated with Triton X-100 [4 % (*v*/*v*)] as positive control.

### Reactive oxygen species and glutathione measurements

Q(N)OH induction of ROSs was determined using a cell-permeable reagent 2′,7′-dichlorofluorescein diacetate dye (DCFDA) in the final concentration of 2 μmol/L (Sigma-Aldrich). For glutathione (GSH) measurement we used 2 μmol/L monochlorobimane (Sigma-Aldrich). Pluronic acid in the final concentration of 16 μmol/L (Sigma-Aldrich) was used as a stabilising agent as described elsewhere (34).

Erythrocytes were isolated and prepared as described above, and following a 1:10 dilution, cell population counted using the TC20™ automated cell counter (Bio-Rad, Hercules, CA, USA). The cells were seeded into a 96-well black plate at a density of 4,000 per well and added Q(N)OHs (IC_25_, IC_50_, or IC_75_) alone or in combination with 1, 10, or 100 μmol/L of fisetin, gently mixed, and immediately inserted into a plate reader (SpectraMax^®^ iD3, Molecular Devices, San Jose, CA, USA). Fluorescence was read continuously at the excitation and emission spectra of 495/529 nm for ROS or 355/460 nm for GSH every 3–10 min over 4 h as described in detail elsewhere (34).

For positive control we used the oxidative stress inducer tBHP at the final concentration of 100 μmol/L. The results are expressed as normalised fluorescence signal ratio to untreated control (which received only the culture medium).

### Superoxide dismutase activity measurement

Superoxide dismutase (SOD) activity was measured with the Superoxide Dismutase Assay Kit (Cayman Chemical, Ann Arbor, MI, USA). After the 4 h treatment described above, erythrocytes were lysed in HPLC-grade water (1:4 dilution) and centrifuged at 10,000 *g* and 4 °C for 15 min. Supernatant was collected and protein concentrations measured using the bicinchoninic acid (BCA) assay (Pierce BCA Protein Assay Kit, Thermo Fisher Scientific, Waltham, MA, USA) as previously described (34). Next, 10 μL of samples and SOD standards per well were mixed with 200 μL of diluted (tetrazolium-based) radical detection solution and 20 μL of diluted xanthine oxidase in a transparent 96-well plate and incubated on a shaker at 22–24 °C for 30 min. Absorbance was measured on an Infinite M200 PRO plate reader (Tecan Austria) at 450 nm. Results are expressed as SOD activity normalised to protein concentration (U/mg of protein).

For positive control we used tBHP at the final concentration of 100 μmol/L.

### Statistical analysis

All data represent the means with the standard error (SE) or standard deviations (SD) of at least two or three independent experiments each done in duplicate or triplicate and were analysed using the Prism 9 software (GraphPad Software, San Diego, CA, USA). Comparisons between the groups were done with the one-way analysis of variance (ANOVA), followed by Dunnett's multiple comparison test. Statistical significance was set to p<0.05 and displayed as follows: & – p<0.05; # – p<0.01; $ – p<0.001; ^*^ – p<0.0001.

## RESULTS

**Figure 3:**
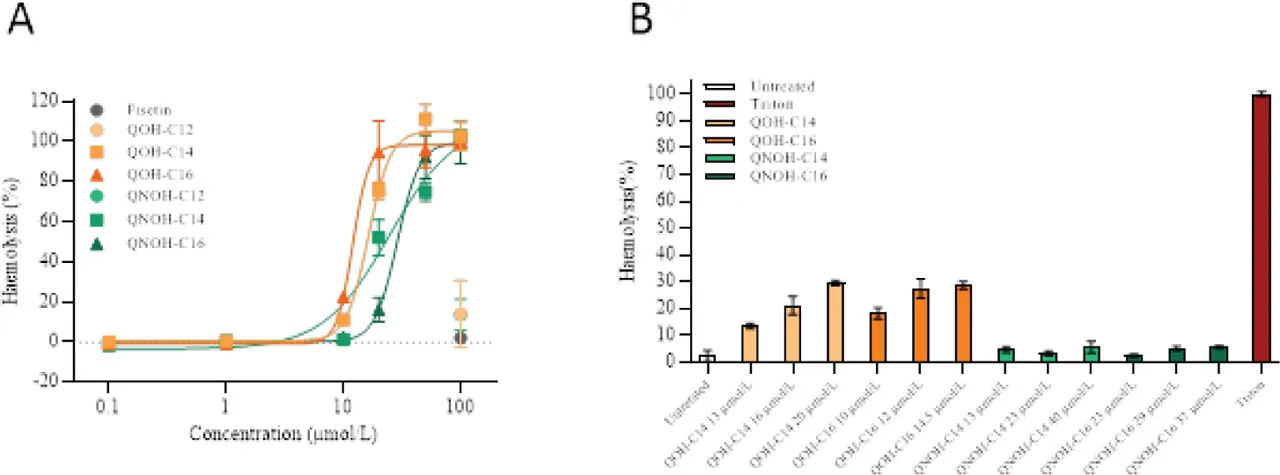
Haemolysis (%) of erythrocytes A) after 24 h exposure to selected Q(N)OHs (0.1– 100 μmol/L) and fisetin (100 μmol/L) and B) after 4 h exposure to Q(N)OHs in determined IC_25_, IC_50_, or IC_75_ compared to untreated control (Triton X-100 was used as positive control)

In the first step of the experiment, involving 24-hour erythrocyte exposure to establish IC_25_, IC_50_, and IC_75_, quinuclidine derivatives with long alkyl chains caused higher haemolysis than those with shorter chains, particularly at concentrations higher than 10 μmol/L (Figure 3). The most toxic compound was QOH-C16, while Q(N) OHs-C12 were not haematotoxic at all (Figure 3A) and were not tested further.

As expected, fisetin caused no haemolysis in the studied concentration range.

**Figure 4:**
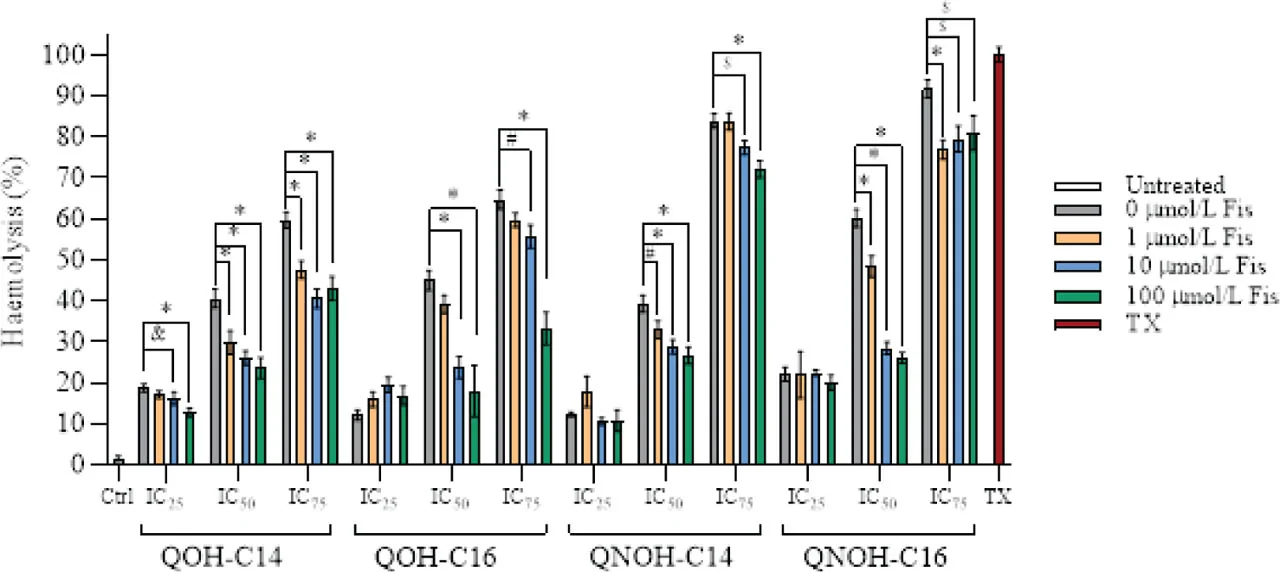
Haemolysis (%) of erythrocytes after 24 h exposure to tested Q(N)OHs-C14 and C16 (in corresponding IC_25_, IC_50_, and IC_75_) in combination with fisetin (Fis; 0, 1, 10 or 100 μmol/L). Untreated control received only culture medium. Triton X-100 (4 %, TX) was used as positive control. Values are presented means ± SD. & – p<0.05; # – p<0.01; $ – p<0.001; * – p<0.0001 compared to untreated control (ANOVA followed by Dunnett's test)

Table 1 shows the obtained IC_25_, IC_50_, and IC_75_ for Q(N) OHs-C14 and C16, which were subsequently used in the 4-hour mechanistic experiments to assess early oxidative responses before the onset of extensive haemolysis with or without fisetin, while the 24-hour haemolysis assay served to confirm the final haematotoxic outcome (Figure 4). As expected, fisetin protected erythrocytes by reducing haemolysis 10–40 %, most effectively at 10 and 100 μmol/L (Figure 4). In addition, for the compounds characterised by very steep concentration-response curves, IC_25_, IC_50_, or IC_75_ concentrations slightly deviated from the expected effect, with differences not exceeding ±15 %.

**Table 1:** IC_25_, IC_50_, IC_75_ values determined from haemolysis curves after 24-hour erythrocyte exposure to Q(N)OHs

**Inhibitory concentrations**	**QOH-C14**	**QOH-C16**	**QNOH-C14**	**QNOH-C16**
IC_25_ (μmol/L)	13	10	13	23
IC_50_ (μmol/L)	16	12	23	29
IC_75_ (μmol/L)	20	14.5	40	37

**Figure 5:**
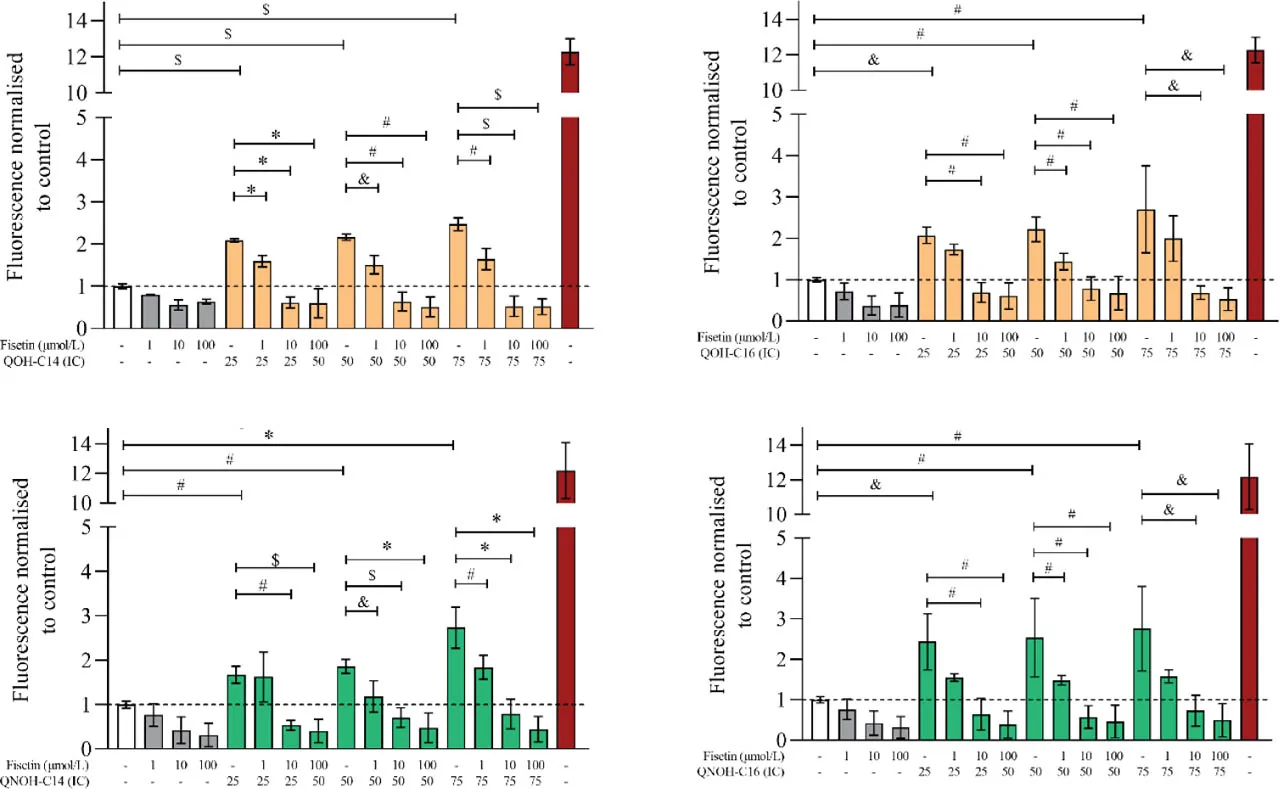
Reactive oxygen species production in erythrocytes exposed to IC_25_, IC_50_, or IC_75_ of Q(N)OH derivatives or their combinations with (1, 10, or 100 μmol/L fisetin over 4 h. Control received only culture medium (white column). *Tert*-butyl hydrogen peroxide (100 μmol/L, red column) was used as positive control. & – p<0.05; # – p<0.01; $ – p<0.001; * – p<0.0001 compared to untreated control (ANOVA followed by Dunnett's test)

Figure 5 shows that quinuclidine derivatives induce significant oxidative stress. ROS levels in erythrocytes exposed to Q(N)OH-C14 and C16 over 4 h rose significantly and plateaued, especially at IC_50_ and IC_75_, indicating maximal oxidative load. Conversely, fisetin co-treatment (10–100 μmol/L) reduced ROS to near-baseline levels, demonstrating a strong antioxidative potential against Q(N)OHs.

**Figure 6:**
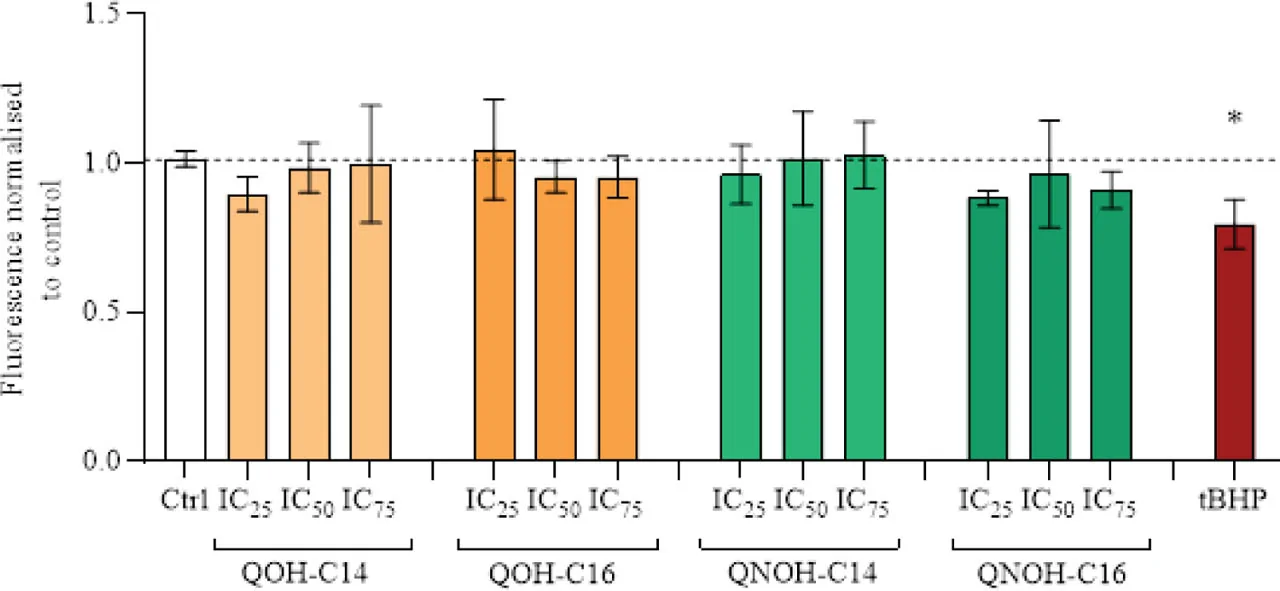
Glutathione (GSH) levels in erythrocytes exposed to IC_25_, IC_50_, or IC_75_ of Q(N)OH derivatives for 4 h. Control received only culture medium (white column). *Tert*-butyl hydrogen peroxide (100 μmol/L, red column) was used as positive control. * p<0.0001 compared to untreated control (ANOVA followed by Dunnett's test)

However, in GSH measurements, fisetin exhibited autofluorescence in the wavelength range used to detect GSH-adducts, rendering GSH quantification of combined treatment void. Figure 6 therefore shows only 4-hour treatment with Q(N)OH derivatives alone. None induced statistically significant changes compared to control.

**Figure 7:**
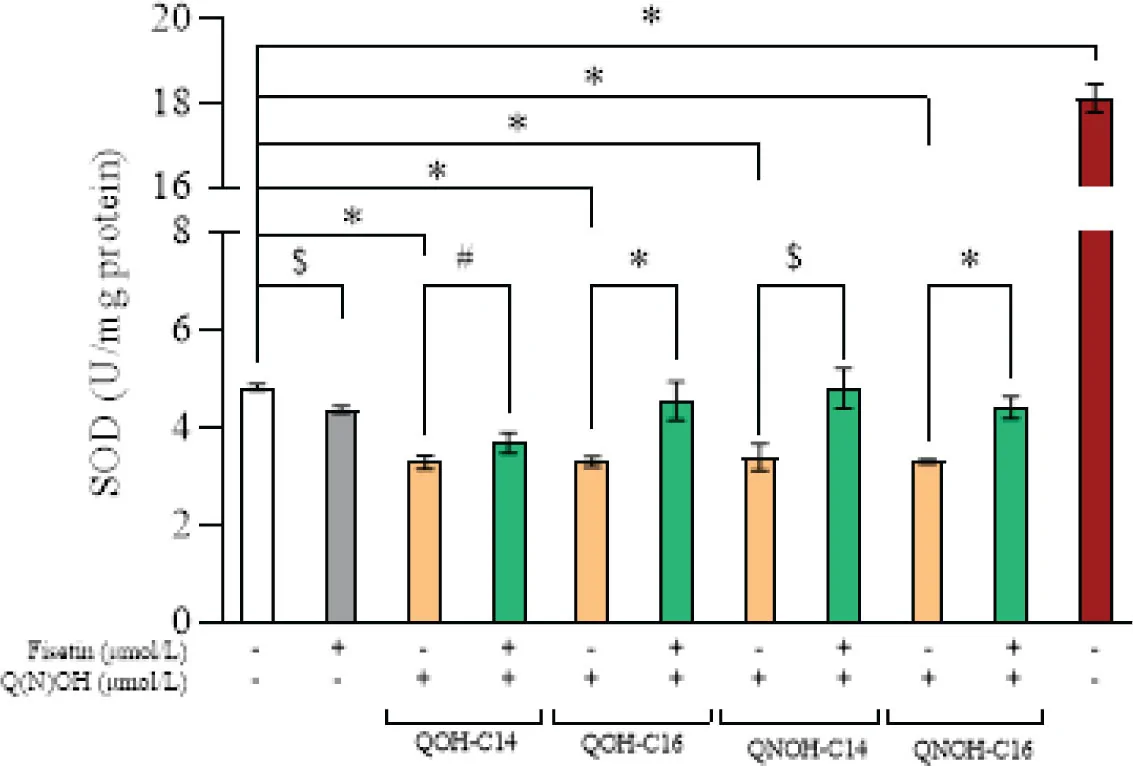
Superoxide dismutase (SOD) activity in erythrocytes exposed to IC_25_, IC_50_, or IC_75_ of Q(N)OH derivatives or their combinations with 10 μmol/L fisetin for 4 h. Control received only culture medium (white column). *Tert*-butyl hydrogen peroxide (100 μmol/L, red column) was used as positive control. & – p<0.05; # – p<0.01; $ – p<0.001; * – p<0.0001 compared to untreated control (ANOVA followed by Dunnett's test)

In line with fisetin's antioxidative effects shown above, Figure 7 shows that its concentration of 10 μmol/L restored SOD activity in erythrocytes treated with IC_50_ of Q(N)OHs for 4 h, particularly in the case of QOH-C16 and QNOH-C16.

## DISCUSSION

Our study confirms that Q(N)OH derivatives with longer alkyl chains (C14 and C16) cause concentration-dependent oxidative injury in erythrocytes, as evidenced by elevated ROS production and haemolysis. These findings are consistent with earlier reports, probably owed to their integration into lipid bilayers, which increases membrane permeability and ROS-mediated lipid peroxidation (1, 6, 35).

Our study also suggests that fisetin can counter these effects by scavenging ROS and supporting cellular antioxidant defences. Besides lowering ROS, it effectively restored SOD activity and attenuated haemolysis in a dose-dependent manner. The protective effects were the most pronounced at 10 and 100 μmol/L, especially against Q(N)OHs at IC_50_. This dose-dependent antioxidant activity aligns with a previous report on fisetin's potent antioxidant activity (36). Unlike flavonoid quercetin, which can act as pro-oxidant at high concentrations or rely on GSH recycling, fisetin can directly scavenge ROS without consuming intracellular GSH (37, 38), which has also been confirmed by our findings. Such a direct mechanism is critical in erythrocytes, which lack the ability to upregulate antioxidant enzyme synthesis (39).

Comparable protective effects have been reported for other flavonoids in erythrocyte models of oxidative injury. For example, flavonols and their glycosides quercetin, myricetin, kaempferol, and rutin were shown to protect human red blood cells from free radical-induced oxidative haemolysis, supporting the concept that polyphenolic compounds can stabilise erythrocyte membranes under oxidative challenge (40). Similarly, orientin and luteolin attenuated oxidative stress in erythrocytes by reducing ROS accumulation and supporting antioxidant defence capacity (41). These findings are in line with our observation that fisetin reduced Q(N)OH-induced ROS generation and haemolysis, suggesting that flavonoid-mediated protection of erythrocytes is not limited to classical oxidants but may also be relevant for membrane-active xenobiotics (40, 41).

The preservation of SOD activity observed in our study is also consistent with previous reports linking flavonoid protection to maintenance of enzymatic antioxidant systems. In AAPH-induced oxidative damage in erythrocytes, flavonoid-rich extracts from mulberry leaves reduced haemolysis and helped maintain antioxidant enzyme activities, including SOD, catalase and glutathione peroxidase (42). Although this model differs from Q(N)OH-induced toxicity, both systems involve oxidative membrane damage and erythrocyte fragility. Therefore, the restoration of SOD activity by fisetin in our study supports the interpretation that attenuation of haemolysis is associated not only with direct ROS scavenging but also with preservation of the endogenous antioxidant defence system (42).

The lack of significant GSH depletion in our model is also relevant. In erythrocytes, GSH represents a key non-enzymatic antioxidant reserve, but these cells have limited capacity to generate new stress-response proteins. Therefore, protection that occurs without measurable GSH depletion suggests that fisetin may reduce the oxidative burden upstream, before extensive consumption of intracellular thiol-based defences occurs. This interpretation is supported by studies in other cell models showing that fisetin can suppress H_2_O_2_-induced ROS accumulation and oxidative cytotoxicity, including in human retinal pigment epithelial cells, where it reduced ROS production and protected against oxidative cell injury (24).

Taken together, our findings fit well with the broader picture showing that flavonoids can protect erythrocytes from oxidative haemolysis, while adding a new and specific context: protection against long-chain quinuclidine derivatives. Unlike previous studies that mainly used classical oxidants such as AAPH, H_2_O_2_ or heat stress, our work demonstrates that fisetin can mitigate oxidative and haemolytic damage caused by pharmacologically active, membrane-interacting AChE inhibitors. This expands the relevance of fisetin from general antioxidant protection towards a potential haemocompatibility-supporting strategy during the development of intravenously administered quinuclidine-based compounds.

## CONCLUSION

The ability of fisetin to reduce haemolysis by 40 % highlights its potential to stabilise erythrocyte membranes under oxidative challenge. Our findings have translational relevance for developing safer intravenous formulations of quinuclidine-based drugs. This is particularly relevant for treating conditions that demand rapid systemic delivery of AChE inhibitors, such as organophosphate poisoning or acute neurotoxic crises. In summary, our data support the hypothesis that quinuclidine-induced erythrocyte toxicity can be effectively mitigated by fisetin co-treatment. By establishing safe concentration thresholds and demonstrating fisetin's protective efficacy, this study offers a framework for further development, including *in vivo* evaluations aimed at safer intravenous therapies involving potent AChE inhibitors.
